# Countdown to 2015: an analysis of donor funding for prenatal and neonatal health, 2003–2013

**DOI:** 10.1136/bmjgh-2016-000205

**Published:** 2017-04-04

**Authors:** Catherine Pitt, Christopher Grollman, Melisa Martínez-Álvarez, Leonardo Arregoces, Joy E Lawn, Josephine Borghi

**Affiliations:** 1 Department of Global Health and Development, London School of Hygiene & Tropical Medicine, London, UK; 2 Maternal, Adolescent, Reproductive, and Child Health (MARCH) Centre, London School of Hygiene & Tropical Medicine, London, UK

## Abstract

**Background:**

In 2015, 5.3 million babies died in the third trimester of pregnancy and first month following birth. Progress in reducing neonatal mortality and stillbirth rates has lagged behind the substantial progress in reducing postneonatal and maternal mortality rates. The benefits to prenatal and neonatal health (PNH) from maternal and child health investments cannot be assumed.

**Methods:**

We analysed donor funding for PNH over the period 2003–2013. We used an exhaustive key term search followed by manual review and classification to identify official development assistance and private grant (ODA+) disbursement records in the Countdown to 2015 ODA+ Database.

**Results:**

The value of ODA+ mentioning PNH or an activity that would directly benefit PNH increased from $105 million in 2003 to $1465 million in 2013, but this included a 3% decline between 2012 and 2013. Projects exclusively benefitting PNH reached just $6 million in 2013. Records mentioning PNH accounted for 3% of the $2708 million disbursed in 2003 for maternal, newborn and child health (MNCH) and increased to 13% of the $9287 million disbursed for MNCH in 2013. In 11 years, only nine records ($6 million) mentioned stillbirth, miscarriage, or the fetus, although the two leading infectious causes of stillbirth were mentioned in records worth $832 million. The USA disbursed the most ODA+ mentioning PNH ($2848 million, 40% of the total) and Unicef disbursed the most ODA+ exclusively benefitting PNH ($18 million, 30%). We found evidence that funding mentioning and exclusively benefitting PNH was targeted to countries with greater economic needs, but the evidence of targeting to health needs was weak and inconsistent.

**Conclusions:**

Newborn health rose substantially on the global agenda between 2003 and 2013, but prenatal health received minimal attention in donor funding decisions. Declines in 2013 and persistently low funding exclusively benefitting PNH indicate a need for caution and continued monitoring of donors' support for newborn health.

**Key questions**

**What is already known about this topic?**

- Progress has been made in reducing the global stillbirth rate and the neonatal mortality rate, but their average annual rates of reduction have lagged behind those for maternal mortality and for under-5 child mortality.
- Previous analyses tracked donor funding for newborns and stillbirth up to 2010 and briefly reported on the 75 Countdown priority countries up to 2012 and on stillbirths globally up to 2013. They found increasing attention for newborn health, but not stillbirths.

**What are the new findings?**

- Using updated data sources and methods, we found that funding mentioning prenatal and neonatal health (PNH) from 52 donors to 156 recipient countries increased substantially between 2003 and 2013 in absolute terms and relative to funding for maternal, newborn and child health (MNCH) as a whole. However, funding mentioning PNH and exclusively benefitting PNH decreased in 2013, even though funding for MNCH increased substantially. Funding exclusively benefitting PNH remained extremely low.
- The USA disbursed the most funding mentioning PNH over the 11-year period ($2848 million, 40%), followed by Canada ($1198 million, 17%) and the International Development Association (World Bank, $585 million, 8%). Unicef disbursed the most funding exclusively benefitting PNH ($18 million), followed by the USA ($15 million) and Japan ($5 million).
- There was some evidence that funding was targeted to countries with greater health and economic need; however, other factors explain much of the substantial variation in funding between recipient countries.
- Babies' prenatal health was rarely mentioned in funding descriptions. Malaria in pregnancy and syphilis were mentioned in funding worth $832 million, but these projects rarely mentioned stillbirth, even though programmes addressing these diseases may make their greatest impact through stillbirth prevention.

**Recommendations for policy**

- Our findings support the importance of global goals for mobilising resources and catalysing change. Implementation and monitoring of the Every Newborn Action Plan and the neonatal target within the Sustainable Development Goals (SDGs), as well as more focus on stillbirths, which still have no SDG target, are essential.
- Effective mechanisms are needed to hold donors to account for high-quality, timely and transparent reporting, and to ensure that data systems are available to support this.

## Introduction

In 2015, 2.6 million babies were stillborn^1^ and a further 2.7 million died as newborns in their first 28 days after birth.^2^ While progress has been made in reducing the global stillbirth rate (SBR) and the neonatal mortality rate (NMR), their average annual rates of reduction have lagged behind those for maternal mortality and for under-5 child mortality, which were targeted in the Millennium Development Goals (MDGs). Between 2000 and 2015, the maternal mortality ratio fell by 37%^3^ while the SBR, which is rarely measured in national statistics and was omitted from the MDGs,^4^ is estimated to have fallen by a more modest 26%.^1^ Over the same period, the postneonatal mortality rate fell by 50%, while the NMR fell by 38%.^2^ By 2015, newborns accounted for 45% of all deaths in children under 5.^2^ Beyond survival, the growing research field of life course epidemiology highlights proliferating evidence on the degree to which fetal and neonatal health affects lifelong health outcomes, notably preventing disability, improving child development and reducing the risk of adult-onset non-communicable diseases.^5^ Promoting the health of babies before, during, and in the first month after birth is therefore an urgent global health challenge, which requires specific attention within the continuum of care and the broader reproductive, maternal, and child health agenda throughout the Sustainability Development Goals (SDG) era.^4^ ^6^

Donor funding and attention may have been among the factors that contributed to the substantial declines in child and maternal mortality.^7^ Between 2003 and 2013, donor funding for child health and for maternal and newborn health increased by 286% and 164%, respectively.^8^ In many low-income and lower-middle-income countries, donor funding constitutes a substantial proportion of overall health spending.^9^ Even in upper-middle-income countries and others where domestic resources account for the vast majority of health expenditure, donor funding may catalyse efforts for issues that might otherwise receive little attention. Estimating the full value of aid that promotes a specific health priority is, however, challenging, because funding directed towards a specific disease or health system challenge will benefit various population groups, not all of which would or could necessarily be described (let alone quantified) in reports of that funding. The value of donor funding specifically mentioning particular issues can, however, serve as a quantitative metric of change in the status of issues on global policy agendas^10^ and can inform estimates of the *minimum* value of aid actually supporting a given health priority. Tracking the value of aid for health priorities is therefore important for holding governments and donors accountable, for assessing the degree to which specific health issues have gained or lost traction on the global agenda, and for indicating whether there may be a substantial mismatch between investment levels and burden of ill health.

In this article, we analyse donor funding for prenatal and neonatal health (PNH) over the period 2003–2013. Following Froen *et al*,^11^ we use the term ‘prenatal health’ to refer to the health of babies before and during birth. Our analysis extends, updates and further analyses previous work to track donor funding for newborns and stillbirths, which we originally conducted for funding up to 2010^12^ and reported on briefly for the 75 Countdown priority countries up to 2012^13^ and globally for stillbirths up to 2013.^11^ In particular, this new analysis explicitly includes neonatal health and stillbirth prevention, involves improved methods for identifying funding mentioning PNH, accounts for unspecified recipients and regional disbursements within country-specific estimates, and is applied to a larger, updated data set for an 11-year period. With these advances, we are now able to better assess trends and the degree to which donor aid was targeted to need.^14^ We compare the value of funding mentioning PNH with estimates of the total value of donor funding benefitting maternal, newborn and child health (MNCH) to indicate whether PNH has gained or lost traction within MNCH and to provide lower and upper bounds for estimates of the total value of donor funding which may actually benefit PNH. Finally, we examine key themes within donors' reporting of their funding mentioning PNH and focus particularly on malaria and syphilis in pregnancy, which are leading infectious causes of miscarriage and stillbirth and contribute to poor neonatal health.^1^

## Methods

### Data source

We identified disbursement records mentioning PNH or directly relevant activities in the Countdown to 2015 ODA+ data set.^8^ ^15^ This data set includes ‘official development assistance’ (ODA) and ‘private grants’, which together we term ‘ODA+’. Other official flows were excluded. Records for the Countdown database were obtained from the Organisation for Economic Co-operation and Development's (OECD) Creditor Reporting System (CRS) aid activity database on 15 January 2015 for the years 2003–2013 and from the Vaccine Alliance (GAVI) on its disbursements between 2003 and 2006, as the latter were missing from the CRS.^15^ Data cover disbursements from 31 high-income donor countries, 20 multilateral institutions, two global health initiatives (GHIs), and one private foundation to 156 recipient countries. Each record contains data on the year, donor, recipient and value of the disbursement, as well as the project title and short and long descriptions of the funded activities. The database avoids double-counting by excluding donor countries' core contributions to multilaterals (only counting these funds at the time they are disbursed for specific activities by the multilaterals) and by attributing donor country funding for a specified project to that donor, even if a multilateral agency is contracted to deliver the project. The Countdown coded each of these records according to an activity-based framework to assess the value of non-research funding for reproductive health, for maternal and newborn health, and for child health. These categories also allow assessment of the value of MNCH and of reproductive, maternal, newborn and child health (RMNCH) as a whole.

### Data coding

We identified records mentioning PNH in the entire Countdown data set using an exhaustive key term search followed by manual review and classification of identified records. We sought to include funding that mentioned the health of the newborn or fetus, or that supported interventions in pregnancy or in the first 4 weeks of life that are proven to improve or maintain the health of the baby before, during, or in the first 28 days following birth. In a previous analysis, we developed key terms by reviewing scientific literature; generating a list of general terms, conditions and diseases, and interventions or programmes meeting our criteria; and then carefully refining our terms.^12^ For example, we sought to include funding mentioning stillbirth, newborns, breastfeeding, or malaria or syphilis in pregnancy, but to exclude funding for antenatal or obstetric care unless it specifically mentioned the baby or PNH interventions. For this analysis, we revised and expanded the previous set of key terms to increase the sensitivity of our search. We conducted the search in Microsoft SQL Server Management Studio (Microsoft Corporation, 2014) using 135 search terms in seven languages (English, French, Dutch, Spanish, Portuguese, Italian, German), and classified each term within a theme (web table 1). We identified those of the 2.1 million records in the Countdown database containing at least one of our search terms in the project title, short description or long description fields reported by donors.

10.1136/bmjgh-2016-000205.supp1 supplementary web table

We exported the subset of records identified by the key terms to Excel for review and classification. Records with a blank or zero disbursement value were excluded. CP and CG read and individually coded the remaining records. First, records that were misclassified (as they did not in any way support or mention prenatal or neonatal health) were removed. Second, records were categorised as supporting either (1) non-research or, (2) research activities; this categorisation was made to allow direct comparisons with the Countdown estimates of ODA+ for RMNCH, which excludes research funding. As the CRS database is a very incomplete source of data on research funding, only those findings regarding non-research funding are presented. Third, records were classified as either (1) exclusively benefiting PNH, or (2) also benefiting other population groups, such as mothers or children older than 1 month. The purpose of this categorisation was to examine the degree of integration of PNH funding within the continuum of care^16^ and to identify the minimum value of funding that could be expected to benefit PNH in practice; both categories are presented in the analysis.

### Data analysis

We present our findings in constant 2013 US$ and assess trends over the 11-year period. We examined variation in funding mentioning PNH over the 11-year period by donor and recipient country. For our main analyses, we present the full disbursement value of all relevant records mentioning PNH and do not make assumptions about the share of funding actually benefiting PNH. We attribute the value of regional and unspecified bilateral disbursements for PNH to recipient countries in proportion to their receipt of country-specific funding for PNH over the entire 2003–2013 period. We present disbursements for each year in total and disaggregated by whether the funding exclusively benefitted PNH or also benefitted other population groups.

We then compare our findings to the Countdown estimates of MNCH funding. Unlike our assessment of PNH, Countdown applied a set of disbursement rules to determine the proportion (0–100%) of the value of each record considered to support MNCH.^8^ ^15^ As a result, for each record mentioning PNH, either the full value of the disbursement or only part of the value of the disbursement may be counted towards the Countdown's estimates of funding for MNCH.^8^

To explore the descriptions of funding mentioning PNH, we classify each record mentioning PNH into at least one of 16 themes, which reflect selected PNH interventions, target populations and causes of ill health.^2^ We assigned records to themes using our main SQL search terms as well as a few additional search terms in Excel (web table 1).

To assess the degree of targeting to recipient countries' need, we produced scatter plots and conducted ordinary least squares regressions. For the scatter plots, we compared total PNH funding and average funding per live birth over the entire 2003–2013 period with the NMR for the 143 of 156 recipient countries for which NMR^17^ and World Bank income group data^18^ were available. We conducted these analyses for all funding mentioning PNH and for funding exclusively benefitting PNH. We used NMR data^17^ for 2008 as the independent variable because the vast majority of PNH funding was disbursed after this date (as we will show), and so we take these data to reflect the information donors had regarding health needs at the time of most of their funding decisions regarding PNH. To estimate average funding per birth over the 11-year period, we divided the total ODA+ for PNH received by each country in each year by the total number of live births^19^ in that country in the same year and then calculated the mean of these ratios over the 11-year period for each country. To illustrate recipient countries' economic needs or capacity to address their own health challenges, we colour-coded each data point by World Bank income group.^18^

With our linear regression models, we sought to determine the degree to which health need (NMR in 2008^17^), economic need (natural logarithm of 2008 per capita gross domestic product (GDP)^18^), and the interaction of these two measures explained variation in our dependent variable, the natural logarithm of cumulative PNH funding over 2003–2013. We specified four models with the outcome defined as the natural logarithm of: total ODA+ mentioning PNH, ODA+ mentioning PNH per live birth, total ODA+ exclusively benefitting PNH and ODA+ exclusively benefitting PNH per live birth.^19^ Both explanatory variables were centred by subtracting the mean value from all observations to facilitate interpretation. We conducted the analyses for the countries in our data set (n=156) for which NMR (n=146), per capita GDP (n=140), and live birth data (if needed) (n=122) were available and for which the specified outcome variable was >0 before the logarithmic transformation. We repeated the analyses of funding per birth for the countries within this set with median populations >250 000 over the period to check whether extremely small countries, all of which received relatively modest absolute levels of funding, affected the observed findings. We also repeated the analyses excluding extreme outliers. To check the appropriateness of our variable transformations, we repeated the analyses without the logarithmic transformations of per capita GDP and of the outcome variables and also by adding a value of one to all outcome values before the logarithmic transformations so as to allow inclusion in the models of countries which received no PNH funding. As all models displayed heteroscedasticity, we used Huber-White estimators to generate robust SEs; other model diagnostics were acceptable. The degree of targeting to need was assessed based on the sign and significance of the coefficients on the independent variables and the R^2^ value for each model.

## Results

Of the 2.1 million records in the Countdown database, 15 062 were identified by our searches as potentially relevant to PNH. After removing 2957 records with blank or zero disbursement values, 955 that were misclassified and 398 supporting PNH research activities, 10 752 non-research records mentioning PNH remained for analysis.

**Figure 1:**
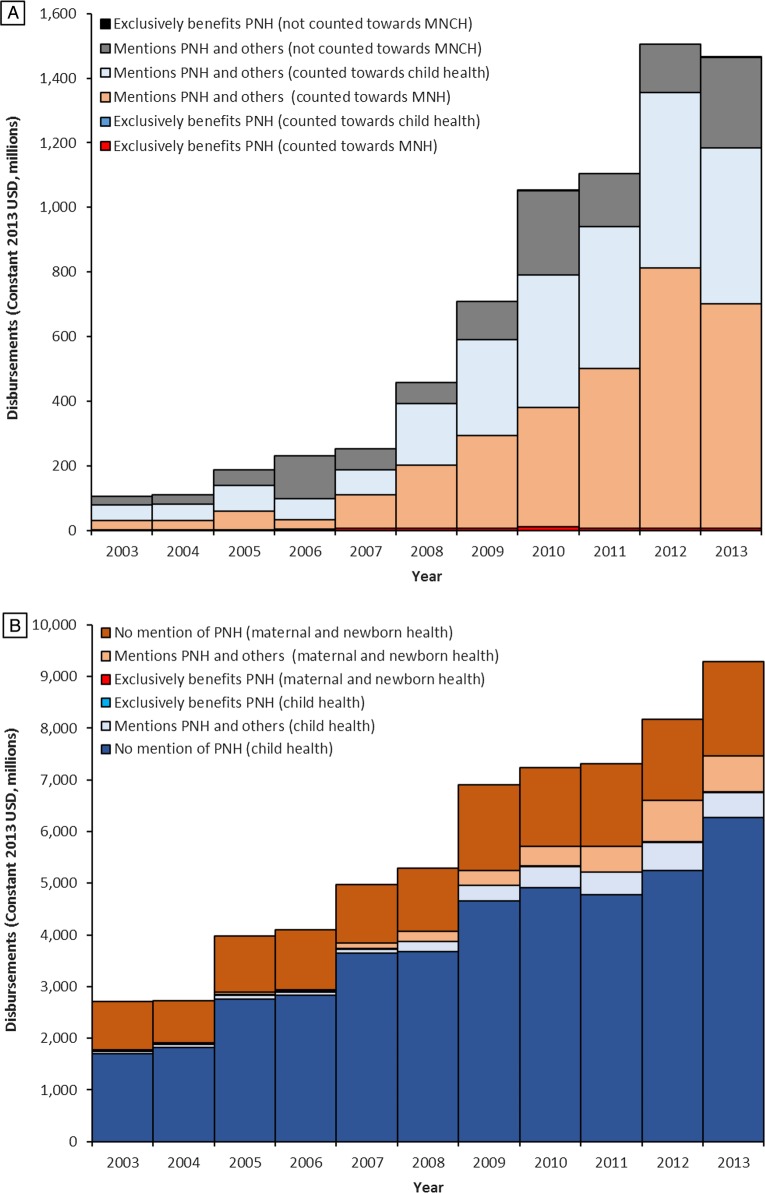
ODA+ for prenatal and neonatal health, 2003–2013, from 54 donors to 156 recipient countries. (A) Presents the full disbursement value of non-research ODA+ mentioning PNH, broken down by whether the funding exclusively benefitted PNH or also benefitted other population groups. It also illustrates how the funding mentioning PNH was categorised within the Countdown framework for estimating the value of funding for maternal, newborn and child health (MNCH). For each record mentioning PNH, between 0% and 100% of the full value of the disbursement may be counted towards the Countdown's estimates of funding for ‘maternal and newborn health’ (MNH) or ‘child health’. (B) presents ODA+ for MNCH based on estimates produced by the Countdown to 2015;^8^ within these estimates are highlighted the value of funding from records which exclusively benefitted PNH and those which mention PNH but also benefit others. ODA+, official development assistance plus private grants; PNH, prenatal and neonatal health.

The annual value of ODA+ mentioning PNH or an activity that would directly benefit PNH increased more than 14-fold from $105 million in 2003 to $1465 million in 2013 (constant 2013 US$, figure 1A, web table 2). ODA+ for PNH increased in every year until 2012 and more than doubled from $708 million in 2009 to a high of $1506 million in 2012, but declined by 3% in 2013.

Almost all ODA+ mentioning PNH also benefitted other population groups; <1% exclusively benefitted PNH over the period and this proportion fell from over 2% in 2003 to <0.5% in 2013. ODA+ exclusively benefitting PNH tripled from just $2 million in 2003 to $6 million in 2013 (figure 1, web table 2). In 2010, this ODA+ exclusively benefitting PNH hit a high of over $11 million, but decreased to $5 million, $7 million and $6 million in the subsequent 3 years.

In figure 1B, we examine ODA+ mentioning and exclusively benefiting PNH within ODA+ for MNCH, which was estimated to have increased nearly 2.5-fold from $2708 million in 2003 to $9287 million in 2013.^8^ As ODA+ exclusively benefitting PNH represented just 0.09% of ODA+ for MNCH over the whole period, it is barely visible in the figure. In contrast, records mentioning PNH and benefitting others accounted for 9% of the total value of aid to MNCH over the period, and increased from 3% in 2003 to 13% in 2013. The value of ODA+ for MNCH that mentioned PNH increased 15-fold between 2003 and 2013, much faster than the 2.5-fold increase in ODA+ for MNCH as a whole. Both values increased in every year from 2003 to 2012; however, from 2012 to 2013 ODA+ for MNCH increased by a further 14%, while the value of ODA+ for MNCH that mentioned PNH fell by 13%. Funding for PNH for the 75 Countdown priority countries showed a similar trend, with an 18-fold increase overall, but a 20-fold increase between 2003 and 2012 and a 9% drop between 2012 and 2013.

Most ODA+ mentioning PNH did not contain any key terms allowing more precise classification of the specific activities, health conditions or level(s) of the health system targeted. (Table 2) More than three-quarters of ODA+ mentioning PNH included generic terms related to newborns, such as ‘neonatal’. Terms specific to breastfeeding were used in descriptions for 12% of the value of ODA+ for PNH; most of these were funded through Unicef and did not mention a specific focus on early, exclusive breastfeeding, which would benefit newborns. Of the 1423 records mentioning breastfeeding, 64 (valued at $15 million across the period) focused exclusively on newborns and mentioned breastfeeding alongside other activities specifically for the newborn. Terms related to neonatal tetanus prevention ($284 million, 4.0%), neonatal resuscitation ($40 million, 0.6%), birth weight ($32 million, 0.5%), preterm birth ($20 million, 0.3%) and umbilical cord care ($15 million, 0.2%) were also mentioned in PNH funding descriptions across the 11 years.

Among the records analysed, only two, valued at $3 million, directly mentioned stillbirth or related terms in its descriptions (table 1). Another project, valued at $24,000 mentioned miscarriage. The fetus was mentioned in six records valued at $3 million. In contrast, malaria in pregnancy, which accounts for 8.2% of all stillbirths (∼220 000 per year) and is the leading cause of stillbirths in sub-Saharan Africa,^1^ was mentioned in projects worth $800 million globally. While all projects mentioning malaria in pregnancy were included in our estimate of ODA+ for PNH, none explicitly mentioned stillbirth, miscarriage or the fetus. Only 38 projects, valued at $31 million, mentioned syphilis, which accounts for 7.7% of global stillbirths; of these, 10 also mentioned newborns, although none mentioned stillbirth, miscarriage or the fetus, even though early fetal deaths and stillbirths from syphilis outnumber neonatal deaths from syphilis by more than two to one.^20^

**Table 1:** Funding for prenatal and neonatal health (PNH) by thematic area, 2003–2013

Theme	Exclusively benefits PNH			Mentions PNH and others			Total mentioning PNH		
	Number of records	Value	Per cent	Number of records	Value	Per cent	Number of records	Value	Per cent
Newborn (generic)	524	53.6	91	8280	5426.0	76	8804	5479.6	76
Breastfeeding	64	14.7	25	1359	859.9	12	1423	874.5	12
Malaria in pregnancy	0	0.0	0	498	800.3	11	498	800.3	11
MNCH	0	0.0	0	305	550.0	8	305	550.0	8
Neonatal tetanus	2	0.6	1	346	283.2	4	348	283.8	4
Postnatal (generic)	0	0.0	0	325	251.6	4	325	251.6	4
Perinatal (generic)	14	5.1	9	169	130.8	2	183	135.9	2
Neonatal resuscitation	64	14.7	25	21	24.8	0	85	39.5	1
Birth weight	63	14.7	25	40	17.7	0	103	32.4	0
Syphilis	0	0.0	0	38	31.5	0	38	31.5	0
Preterm birth	71	15.5	26	9	4.7	0	80	20.2	0
Umbilical cord	64	14.9	25	3	0.1	0	67	15.0	0
Fetus (generic)	2	0.2	0	4	2.7	0	6	2.9	0
Stillbirth	1	0.5	1	1	2.4	0	2	2.9	0
Kangaroo mother care	0	0.0	0	2	1.2	0	2	1.2	0
Birth asphyxia	0	0.0	0	0	0.0	0	0	0.0	0
Jaundice	0	0.0	0	0	0.0	0	0	0.0	0
Miscarriage	0	0.0	0	1	0.0	0	1	0.0	0
Total	539	58.8	100	10 213	7116.7	100	10 752	7175.5	100

Total value of funding (constant 2013 US$ millions) provided over the period 2003–2013. Themes are not mutually exclusive, so columns sum to more than 100%. Search terms used to classify records into themes are provided in web table 1.

MNCH, maternal, newborn and child health (this category reflects those projects identified by this abbreviation or its equivalent in other languages).

Over the 11-year period, two donors together accounted for 56% of all ODA+ mentioning PNH: the USA ($2848 million, 40%) and Canada ($1198 million, 17%). They were followed by the World Bank's International Development Association (IDA, $585 million, 8%), the Global Fund ($522 million, 7%) and the UK ($453 million, 6%) (table 2). The leading donors of ODA+ exclusively benefitting PNH were Unicef ($18 million, 30%), the USA ($15 million, 25%), Japan ($5 million, 8%) and the UK ($4 million, 6%). All ODA from Canada, the IDA and the Global Fund that mentioned PNH supported activities that would also benefit other population groups, as did 99% of ODA from the UK and the USA. Canada provided nearly all of its funding mentioning PNH in 2011–2013, when it disbursed $301 million, $282 million and $286 million, respectively. The UK's funding increased substantially in 2012 and 2013, when it disbursed $180 million and $132 million, respectively. Together, bilateral donors accounted for 66% of ODA+ exclusively benefitting PNH and 74% of funding also benefitting others. The UK, Sweden and Australia all substantially increased their ODA+ mentioning PNH in 2012 but then decreased their funding in 2013 by a combined total of $107 million, which more than accounted for the overall $40 million decline in ODA+ mentioning PNH in 2013.

**Table 2:** Funding mentioning prenatal and neonatal health (PNH) by donor, 2003–2013

Rank	Donor	Exclusively benefits PNH	Mentions PNH and others	Total
1	USA	14.7	2833.8	2848.5
2	Canada	0.0	1198.3	1198.3
3	World Bank International Development Association	0.0	584.9	584.9
4	Global Fund for AIDS, Tuberculosis and Malaria	0.0	522.2	522.2
5	UK	3.6	449.2	452.9
6	Australia	0.5	193.8	194.3
7	Bill & Melinda Gates Foundation	2.2	191.5	193.8
8	UN Population Fund (UNFPA)	0.0	169.5	169.5
9	Asian Development Bank Special Fund	0.0	157.6	157.6
10	Norway	1.0	112.5	113.5
11	Sweden	0.0	102.9	103.0
12	European Union Institutions	0.1	92.5	92.6
13	UN Children's Fund (Unicef)	17.7	68.8	86.6
14	The Vaccine Alliance (GAVI)	0.0	66.7	66.7
15	Spain	3.8	55.1	58.9
16	Japan	4.7	51.0	55.7
17	Netherlands	0.0	49.5	49.5
18	France	0.1	45.0	45.1
19	Switzerland	3.4	34.5	37.9
20	Korea	1.0	24.6	25.7
21	Belgium	1.9	21.9	23.8
22	Germany	0.2	21.8	22.1
23	World Bank International Development Bank Special Fund	0.0	14.6	14.6
24	Luxembourg	1.2	10.8	12.0
25	Italy	2.3	6.6	8.9
26	Denmark	0.0	8.4	8.4
27	WHO	0.0	6.5	6.5
28	New Zealand	0.0	5.6	5.6
29	OPEC Fund for International Development	0.0	5.2	5.2
30	Finland	0.0	4.2	4.2
31	Iceland	0.0	2.4	2.4
32	UNAIDS	0.0	1.4	1.4
33	United Arab Emirates	0.0	1.3	1.3
34	Portugal	0.0	0.8	0.8
35	Ireland	0.0	0.7	0.7
36	Greece	0.2	0.0	0.2
37	Austria	0.0	0.2	0.2
38	Czech Republic	0.0	0.2	0.2
39	Poland	0.0	0.2	0.2
40	Slovak Republic	0.0	0.1	0.1
41	UN Development Programme (UNDP)	0.0	0.1	0.1
	Total	58.8	7116.7	7175.5

The table presents the total value of non-research funding (constant 2013 US$ millions) mentioning PNH provided by each donor over the period 2003–2013, by whether the funding exclusively benefitted PNH or also benefitted other population groups. Donors are ranked from highest to lowest total disbursements. Of the 54 donors in the Countdown database, 13 did not provide any non-research funding mentioning PNH in 2003–2013 period and so do not appear in this table: Estonia, Kuwait, Slovenia, African Development Bank, African Development Fund, Arab Fund for Economic and Social Development, Arab Fund for Economic Development in Africa (BADEA), Global Environment Facility, United Nations Refugee Agency (UNHCR), United Nations Peacebuilding Fund, International Monetary Fund (Concessional Trust Funds) and the World Food Programme. Donors appearing in the table with a total disbursement showing as ‘$0.0 million’ disbursed <$50 000, which was rounded down. There is no double-counting of bilateral and multilateral ODA and private grants, so the columns sum to 100%.

OPEC, Organization of the Petroleum Exporting Countries; PNH, prenatal and neonatal health; UN, United Nations.

**Figure 2:**
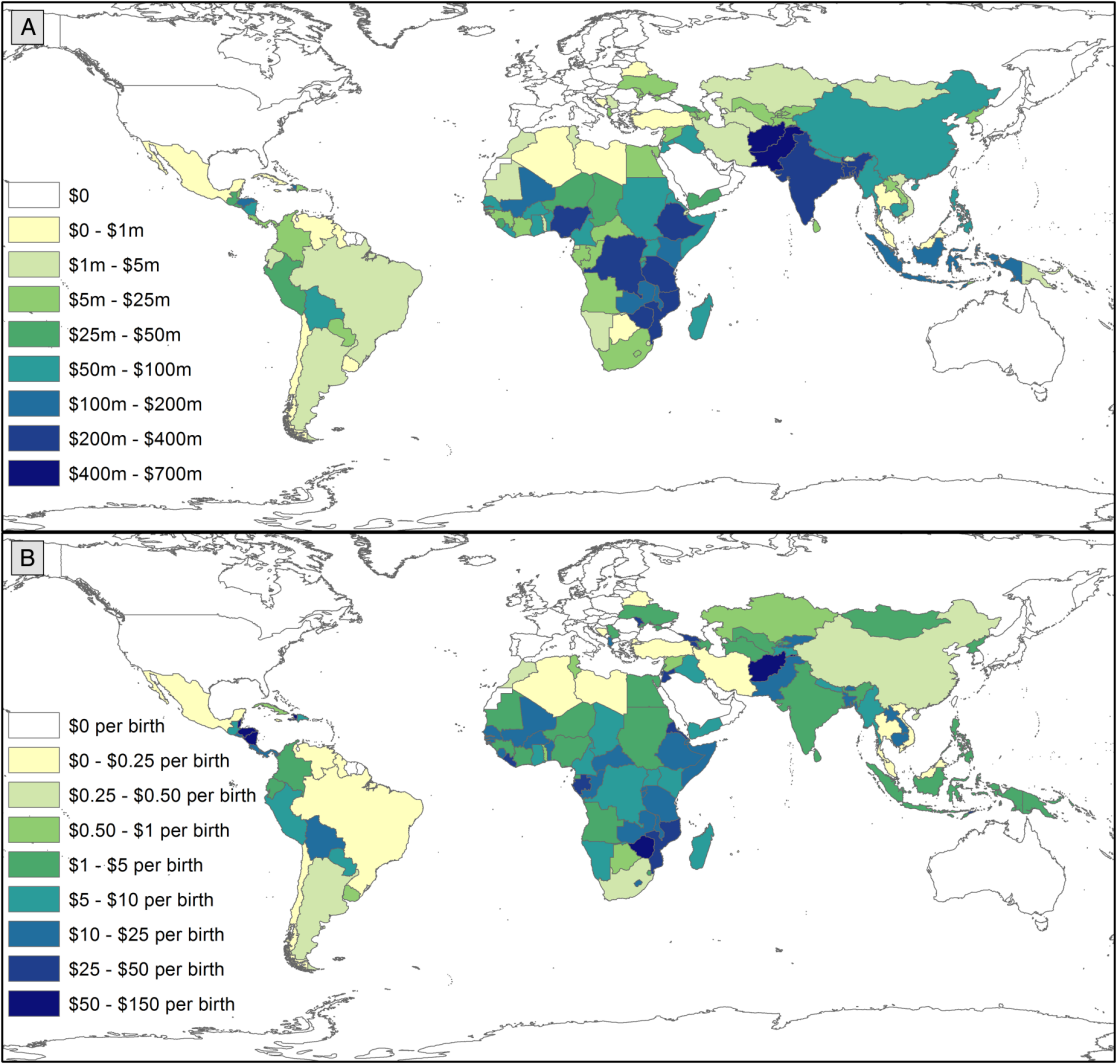
Maps: Variation by recipient country in ODA+ mentioning PNH in total and per live birth, 2003–2013. (A) Presents variation in total ODA+ mentioning PNH by recipient country. (B) Presents variation in ODA+ mentioning PNH per birth by recipient country. Data are presented in constant 2013 US$ and were prepared in ArcGIS V.10.3. ODA+, official development assistance plus private grants; PNH, prenatal and neonatal health.

**Figure 3:**
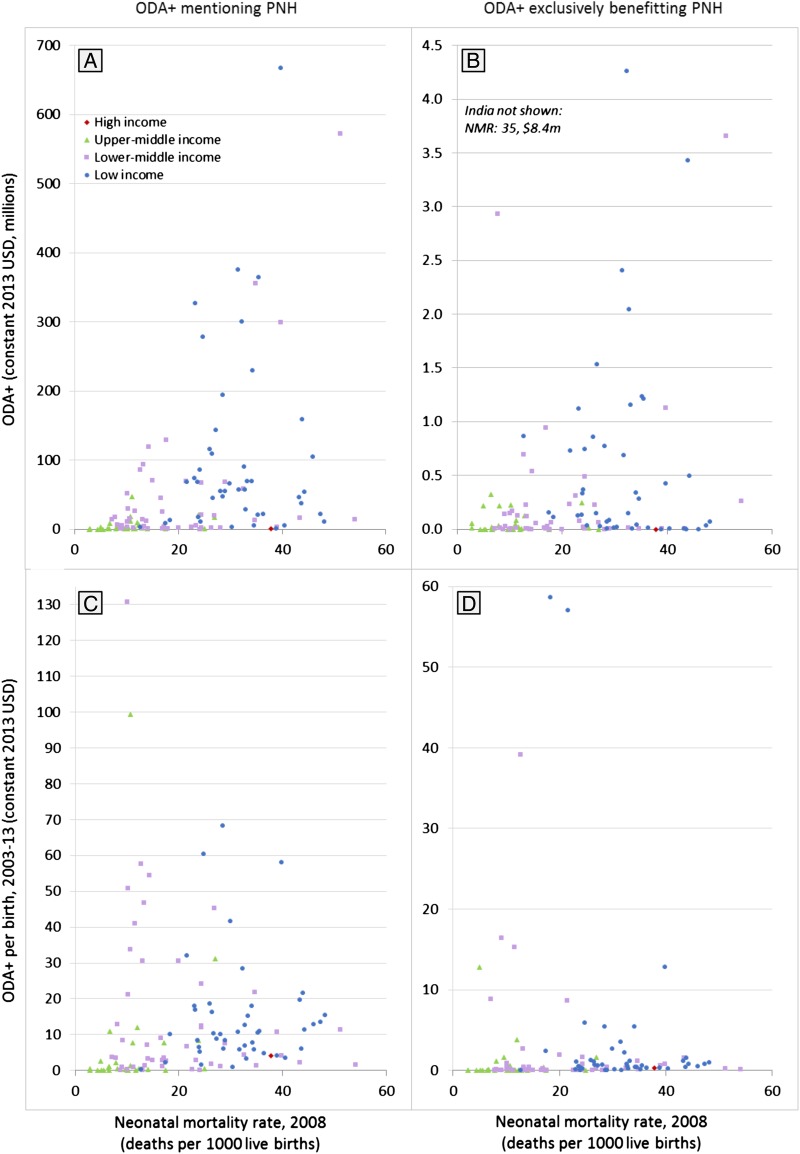
Targeting of ODA+ mentioning prenatal and neonatal health (PNH) to countries with the greatest need, 2003–2013. The four scatter plots present the association between the amount of official development assistance and private grants (ODA+) received by each country and the neonatal mortality rate (our metric of health needs) in that country. Data points are colour-coded by country income group to provide an indication of their economic need. The four scatter plots show total funding over the 11-year period (plots A and B) and average ODA+ per birth (plots C and D) that mentions PNH (plots A and C) and that exclusively benefits PNH (plots B and D).

Funding for PNH also varied substantially by recipient country (figures 2 and 3, web tables 3 and 4). All 156 recipient countries in the Countdown ODA+ database received funding supporting MNCH, while 125 received ODA+ mentioning PNH and 100 received funding exclusively benefitting PNH. The 75 Countdown priority countries together received 91% of all ODA+ for PNH and this proportion remained relatively consistent over the period. The recipients of the most ODA+ mentioning PNH were Afghanistan ($667 million including regional allocations), Pakistan ($572 million), Bangladesh ($375 million), Ethiopia ($364 million) and India ($356 million). Taking population sizes into account, the leading recipients of ODA+ for PNH per birth tended to be much smaller countries: Belize ($131 per birth), Fiji ($99 per birth), Haiti ($68 per birth), Zimbabwe ($60 per birth), Afghanistan ($60 per birth) and Nicaragua ($58 per birth). India received $8 million in ODA+ exclusively benefitting PNH, followed by Mozambique ($4.3 million), Pakistan ($3.7 million) and Mali ($3.4 million); only 14 countries received more than $1 million for projects exclusively benefitting PNH across the period. With $1.08 per birth, the West Bank and Gaza Strip received the largest volume of funding exclusively benefitting PNH, but could not be included in our scatter plots or regressions because NMR data was unavailable. It was followed by Laos ($0.62 per birth), Ukraine ($0.60 per birth), Uruguay ($0.58 per birth) and Mali ($0.46 per birth).

Funding mentioning PNH was somewhat targeted to countries with greater need, but there was less evidence that funding exclusively benefitting PNH was targeted to need (web table 5). The four scatter plots show the weak association between our metric of health need, NMR, and the value of funding mentioning PNH and exclusively benefitting PNH in total and per birth (figure 3). The multivariable regression analyses indicated that countries with lower per capita GDP and higher NMR tended to receive more ODA+ mentioning PNH; however, while the negative relationship with per capita GDP was strongly significant in the total ODA+ (p<0.001) and per birth (p<0.001) models, the positive relationship with NMR was only weakly significant in the per birth model (p=0.058) and not significant in the total ODA+ model (p=0.107). Both models demonstrated high goodness of fit, although total ODA+ mentioning PNH appeared better targeted to health and economic need (R^2^=0.47) than ODA+ per birth (R^2^=0.35). For funding exclusively benefiting PNH, there was less evidence of targeting to need: the negative association with per capita GDP was strong in both the total ODA+ model (p=0.021) and the per birth model (p<0.001), but the association with NMR was not significant and negative in the total ODA+ model (p=0.664) and weak and positive in the per birth model (p=0.054). The goodness of fit was poor in the total ODA+ model (R^2^=0.08) but reasonable in the per birth model (R^2^=0.29) and both were lower than in the models of ODA+ mentioning PNH. When we reran the analyses under alternative model specifications, the significant, negative relationship with per capita GDP remained in all models, as did the lack of significant relationship between NMR and total ODA+ mentioning or exclusively benefitting PNH; however, some specifications of the per birth models indicated a moderately significant, positive relationship with NMR while other specifications indicated that the relationship was not significant (web table 5).

## Discussion

We found substantial increases in the value of donor funding mentioning PNH between 2003 and 2013 in absolute terms and relative to funding for MNCH as a whole. We interpret these findings as reflecting a substantial rise in attention for newborn health on the global agenda, particularly from 2008. Nonetheless, funding exclusively benefitting PNH remained extremely low, reaching just $6 million in 2013 of a total of more than $9 billion disbursed for MNCH. From 2012 to 2013, funding mentioning PNH and exclusively benefitting PNH decreased, even though funding for MNCH increased by 14%. The USA and Canada provided over half of funding mentioning PNH over the period; with the rise to power of Donald Trump and the departure of Prime Minister Stephen Harper, who personally championed RMNCH within the G8, this funding may be subject to political changes. We argue that these findings indicate a need for caution and continued monitoring of donors' support for newborn health. Consistent with previous findings,^11^ ^12^ we found that prenatal health, including stillbirth, continued to receive minimal attention in donors' descriptions of their programmatic funding relative to its burden. Some donors have explicitly prioritised PNH to a greater extent than others; the list of leading donors mentioning PNH in their funding records shares commonalities with, but is by no means the same as, the leading donors for RMNCH as a whole.^8^ Canada, in particular, is much more highly ranked for PNH funding than for overall RMNCH funding, although none of its funding exclusively benefited newborn health and stillbirth prevention. Most Countdown priority countries received <$1 million in funding even mentioning PNH across the entire 11-year period. We found evidence of targeting of funds mentioning PNH to countries with lower GDP per capita, but there was inconsistent evidence of targeting based on NMR, and less evidence that funding exclusively benefitting newborns was targeted to need.

The actual value of funding that ultimately benefits PNH could be nearly as low as the value of funding exclusively benefitting PNH ($6 million in 2013) or nearly as high as the value of funding benefitting MNCH as a whole ($9287 million in 2013). While much of the funding for maternal health could and should also benefit PNH even if PNH is not mentioned directly, the slower progress in reducing neonatal mortality and stillbirth rates relative to postneonatal and maternal mortality indicates that the benefits to PNH from maternal and particularly from child health funding cannot be assumed.^6^ Funding focused on PNH may therefore play an important role in ensuring that the 5.3 million stillbirths and neonatal deaths receive due attention within programmes. Funding mentioning PNH that is also intended to benefit other population groups may be more likely to benefit PNH than maternal and child health programmes which do not mention PNH, but this is not clear, especially for those where the mention is just part of the term ‘maternal, newborn and child health’. In practice, it is uncertain whether babies actually benefit from maternal and child health programmes and even wider primary healthcare, hospital and health systems support, regardless of whether PNH is mentioned in funding summaries. In some programmes, implementers may be able to influence the focus of broader investments, but in others, such as large immunisation programmes, the investments are very fixed. For example, even investments in care at birth or more midwives would not necessarily reduce stillbirths or neonatal deaths if key skills or equipment, such as fetal monitoring during labour and neonatal resuscitation are not included. Conversely, programmes addressing malaria or syphilis in pregnancy rarely mention stillbirths and yet may make their greatest impact through stillbirth prevention. We also have no information on the relative efficiency with which funds are used and translated into population-level benefits in different settings and over time.

While we found an 18-fold increase in the value of MNCH funding mentioning PNH between 2003 and 2012 for the Countdown priority countries, this is only half the 34-fold increase previously reported for the same period.^13^ In contrast, we found a 10-fold increase in the total value of funding mentioning PNH for all recipients between 2003 and 2010, which is slightly higher than the nearly ninefold increase previously reported for that period.^12^ Most of the changes in our estimates from those previously published reflect donors adding retrospective data, which led to increases in their funding estimates for past years. Several of our methodological improvements, notably our inclusion of a relevant proportion of regional and unspecified funding within country-specific estimates, led to increases relative to previous estimates; however, the effects of these changes were eclipsed by donors' retrospective amendments to their data. While such amendments should be encouraged, they underscore the importance of holding donors to account for high-quality, timely and transparent reporting^21^ and of ensuring that data systems are available to support this.

The CRS database—on which the Countdown to 2015 ODA+ is based—has several advantages, including its consistent and comparable reporting framework, which avoids double-counting of funds disbursed from countries to multilateral institutions and global health initiatives, as well as the fact that the donors themselves report and agree all funding reported. Nonetheless, the CRS also has a number of disadvantages, notably the lack of reports from China, Brazil, some other donor countries, and all but one private foundation, and its sector and purpose code classification system, which does not facilitate the identification of funding for different types of health activities, beneficiary groups, or health conditions, such as PNH.^12^ Our approach of first implementing a sensitive key term search and then individually reading and coding projects for this analysis was both efficient and, we believe, a reasonably accurate and precise method for maximally exploiting the available data and overcoming some of the serious limitations of the sector and purpose code framework in the CRS database. Such combinations of automated and human coding could prove a useful approach to enable aid tracking for specific issues and should be considered as new mechanisms are established to hold donors accountable for their obligations and commitments in the SDG era.^22^

These findings must therefore be interpreted with the caveats given; however, they have important policy implications. Our findings build on our past analyses,^12^ ^13^ providing further quantitative evidence of the rise of newborn health on the global agenda, and the continued neglect of stillbirths and prenatal health in programmatic funding more generally.^4^ ^11^ Future research should examine why some countries receive so much more funding mentioning PNH than others and whether differences in funding descriptions are associated with differences in programme implementation. We argue that our findings support the importance of global goals for mobilising resources and attention and catalysing change. Implementation and monitoring of the Every Newborn Action Plan^23^ and the neonatal target within the SDGs, as well as more focus on stillbirths, which still have no SDG target, are essential,^4^ especially in light of the apparent reductions in 2013.
