# Supplementary material for: Countdown to 2015: an analysis of donor funding for prenatal and neonatal health, 2003–2013
Source: BMJ Glob Health. 2017 Apr 4;2(2):e000205. doi: 10.1136/bmjgh-2016-000205 (PMC5435259; doi:10.1136/bmjgh-2016-000205)
Supplement: supplementary web table [file bmjgh-2016-000205supp_tables.pdf]

Countdown to 2015: An analysis of donor funding for prenatal and neonatal health, 2003-2013

Catherine Pitt, Christopher Grollman, Melisa Martinez-Alvarez, Leonardo Arregoces, Joy E Lawn, Josephine Borghi

Department of Global Health and Development, London School of Hygiene & Tropical Medicine, 15-17 Tavistock Place, London WC1H 9SH, United Kingdom (Catherine Pitt, assistant professor, Christopher Grollman, research fellow, Melisa Martinez-Alvarez, assistant professor, Leonardo Arregoces, research fellow, Josephine Borghi, associate professor)

Maternal, Adolescent, Reproductive, and Child Health (MARCH) Centre, London School of Hygiene & Tropical Medicine, Keppel Street, London WC1E 7HT, United Kingdom (Joy E Lawn, professor)

Correspondence to: catherine.pitt@lshtm.ac.uk

SUPPLEMENTARY TABLES

Supplementary table 1: Search terms
Supplementary table 2: Funding mentioning and exclusively benefiting PNH by year, 2003-13
Supplementary table 3: Funding mentioning PNH by recipient country
Supplementary table 4: Regional funding mentioning PNH by recipient region
Supplementary table 5: Results of regressions assessing the degree of targeting of PNH funding to health and economic need

Supplementary table 1: Search terms

The search terms listed below were used to conduct two searches:

First, a search was implemented in the entire Countdown to 2015 ODA+ Database to identify all records mentioning PNH; this search was implemented in Microsoft SQL using the search terms listed in column 2 below. In SQL, searches are not case sensitive and "%" identifies any number of characters greater than or equal to 0. Square brackets identifyany of the characters listed within them; for example, searching for "%N[eé]ONAT%" is equivalent to two separate searches for "%NeONAT%" or "%NéONAT%".

Second, once the set of correctly classified, non-research records mentioning PNH were identified in Excel, each of these records was grouped into at least one theme. First, each record was grouped into all the themes associated with the SQL search terms which had identified it in the first search. Then, additional searches were conducted in Excel to classify records into additional themes; these are presented in column 3 below. For some themes, such as congenital syphilis, the original search terms were sufficiently sensitive. For other themes, additional search terms were needed because the original search terms did not include terms specific to conditions or interventions that would invariably also use a more generic term related to PNH. For example, records mentioning neonatal resuscitation were identified as relevant to PNH by the term "neonatal", and so to classify records into this theme we also searched within our set of records relevant to PNH for the term "resuscit". Similarly, we also generated additional search terms to identify additional records relevant to two further themes, neonatal tetanus and malaria in pregnancy In Excel, searches are not case sensitive and will identify the given text string occurring anywhere within the fields searched.

**Theme**	**Search terms used to identify records relevant to PNH in SQL**	**Additional search terms added to classify relevant PNH records by theme in Excel**
Newborn (generic)	%N[eé]ONAT%<br>%N[eé]O-NAT%<br>%NEONAAT%<br>%NEUGEBOREN%<br>%NEWBORN%<br>%NOUVEAU[-]N[eé]%<br>%PASGEBOREN%<br>%RECEM[-]NASCIDO%<br>%RECI[eé]N NACIDO%	
MNCH	%IMNCI%<br>%MNCH%<br>%MNH%<br>%PCIMNI%<br>%SMN%<br>%SMNE%	
Breastfeeding	% ALLAITE%<br>% LACTANC%<br>%ALLATTA%<br>%AMAMANT%	

	%BORSTVOED%<br>%BREAST[-]FE%<br>%BREASTFE%<br>%FRUHES STILLEN%<br>%LAKTAT%<br>%LATTAZIONE%<br>ALLAITE%<br>LACTANC%	
Malaria in pregnancy	%INTERMITTENT PRESUMPTIVE TREATMENT%<br>%INTERMITTENT PREVENTIVE TREATMENT%PREGNAN%<br>%IPTP%<br>%MAL[Aá]RIA % GR[Aá]VID%<br>%MALARIA % EMBARAZ%<br>%MALARIA % GR[Aá]VID%<br>%MALARIA IN PREGNAN%<br>%MALARIA IN%ZWANGER%<br>%MALARIA% SCHWANGER%<br>%PALUDI% EMBARAZ%<br>%PALUDI% ENCEINT%<br>%PALUDI% GR[Aá]VID%<br>%TPI%<br>%TRAITEMENT PR[eé]SOMPTIF INTERMITTENT%<br>%TRAITEMENT PR[eé]VENTIF INTERMITTENT%<br>%TRATAMENTO INTERMITENTE PREVENTIVO%<br>%TRATAMIENTO PREVENTIVO INTERMITENTE%EMBARAZ%	malaria<br>paludi
Neonatal tetanus	% TT%<br>%ANATOXIN%<br>%TETANIQUE%<br>%TETANUSIMPFUNG%<br>%TOXOID%<br>TT%	tetanus
Postnatal (generic)	%POS[-]NATA%<br>%POST[-]NAT%<br>%POSTNAT%<br>%WOCHENBETT%	
Perinatal (generic)	%P[eé]RINAT%	
Fetus (generic)	% FETAL%<br>% FETO%<br>% FETUS%<br>% FOETAL%<br>%FOETAAL%<br>%FOETUS%<br>FETAL%<br>FETO%<br>FETUS%<br>FOETAL%	
Umbilical cord	% CORDON%<br>%CORD CARE%<br>%NABELINFEKTION%<br>%NABELPFLEGE%	

	%NAVELSTRENG%<br>%OMB[ei]LICAL%<br>%UMBIGO%<br>%UMBILIC%	
Preterm birth	%ANTENATAL STEROID%<br>%FRUHGEBURT%<br>%NASCIDO MUITO CEDO%<br>%PR[eé]MATUR%<br>%PREMATUUR%<br>%PRETERM%<br>%UNREIF%<br>%VROEG GEBOORTE%	
Birth weight	%BAIXO PESO%<br>%BAJO PESO%<br>%BIRTH_WEIGHT%<br>%BIRTHWEIGHT%<br>%FAIBLE POIDS%<br>%GEBOORTE GEWICHT%<br>%GEBOORTEGEWICHT%<br>%GEBURTSGEWICHT%<br>%PESO DE NACIMIENTO%<br>%PESO AL NACER%<br>%POIDS DE NAISSANCE%<br>%POIDS [Aà] LA NAISSANCE%<br>%SOTTOPESO%<br>%UNTERGEWICHT%	
Neonatal resuscitation		resuscitat
Stillbirth	%DOODGEBOR%<br>%DOOGEBOR%<br>%MORT[-]N[eé]%<br>%MORTINAISSANCE%<br>%MORTINATALIDAD%<br>%MORTINATO%<br>%NACE%MUERTO%<br>%NACIDO MUERTO%<br>%NASCE%MORTO%<br>%NATIMORTALIT%<br>%NATIMORTO%<br>%NATO MORT%<br>%STILLB%<br>%TOTGEB%	
Syphilis	%LUES CONNATA%<br>%S[ií]FILIS%<br>%SIFILIDE%<br>%SYPHILIS%	
Kangaroo Mother Care	%CANGU%<br>%CANGURO%<br>%CANGURU%<br>%HAUTKONTAKT%<br>%KANGARO%	

	%KANGOUROU%<br>%KANGURU%<br>%PEAU___PEAU%<br>%PELE A PELE%<br>%PELLE A PELLE%<br>%PIEL CON PIEL%<br>%SKIN_TO_SKIN%	
Birth asphyxia	%ASFISSIA%<br>%ASPHYXI%	
Jaundice	%GELBSUCHT%<br>%ICTER[ií]CIA%<br>%IKTERUS%<br>%ITTERIZIA%<br>%JAUNDICE%<br>%JAUNISS%	
Miscarriage	%ABORTO ESPONT%<br>%ABORTO SPONT%<br>%AVORTEMENT SPONT%<br>%FEHLGEBURT%<br>%MISCARRIAGE%<br>%MISKRAAM%<br>%SPONTANE AFBREKING%	

Supplementary table 2: Funding mentioning and exclusively benefiting PNH by year, 2003-13

Total value of ODA+ for PNH (constant 2013 USD, millions) disbursed over the period 2003-13 disaggregated by year and by whether the funding exclusively benefitted PNH or also benefitted other population groups.

	2003	2004	2005	2006	2007	2008	2009	2010	2011	2012	2013	TOTAL
**TOTAL funding mentioning PNH**	**104.9**	**109.4**	**187.2**	**230.8**	**252.3**	**457.3**	**707.9**	**1050.6**	**1104.3**	**1505.5**	**1465.4**	**7175.5**
Funding exclusively benefitting PNH	2.1	2.5	2.4	3.9	5.9	6.2	5.7	11.4	5.4	6.9	6.4	58.8
Funding mentioning PNH but also benefitting other population groups	102.7	106.9	184.8	226.9	246.3	451.1	702.2	1039.3	1098.9	1498.6	1459.0	7116.7

Supplementary table 3: Funding mentioning PNH by recipient country

Total value of funding (constant 2013 USD, millions) received by each country over the period 2003-13 by whether the funding exclusively benefitted PNH or also benefitted other population groups. The value of funding for regional and unspecified recipients which we attribute to each individual country is also reported. Recipients are ranked from highest to lowest cumulative disbursements.

Country	Not research		Total country-specific	Regional allocation	Total including regional allocation
	Exclusively benefits PNH	Mentions PNH and others			
**TOTAL**	**50.6**	**5987.2**	**6037.8**	**1137.7**	**7175.5**
Afghanistan	0.4	589.7	590.1	76.4	666.5
Pakistan	3.7	502.9	506.5	65.6	572.1
Bangladesh	2.4	330.0	332.4	43.0	375.4
Ethiopia	1.2	293.8	295.0	69.2	364.2
India	8.4	306.4	314.8	40.7	355.5
Tanzania	1.1	263.5	264.7	62.1	326.8
Mozambique	4.3	239.2	243.5	57.1	300.6
Nigeria	1.1	241.0	242.1	56.8	298.9
Zimbabwe	0.0	225.0	225.1	52.8	277.9
Democratic Republic of the Congo	0.0	185.3	185.4	43.5	228.9
Haiti	0.0	157.8	157.8	36.1	193.9
Mali	3.4	125.5	128.9	30.3	159.2
Kenya	0.0	115.6	115.6	27.1	142.7
Indonesia	0.0	113.4	113.4	15.8	129.2
Honduras	0.5	99.0	99.6	18.8	118.3
Zambia	0.9	92.6	93.4	21.9	115.3
Malawi	0.1	88.4	88.6	20.8	109.4
South Sudan	0.0	84.8	84.8	19.9	104.7
Jordan	0.1	82.5	82.6	10.8	93.4
Burkina Faso	0.1	73.2	73.3	17.2	90.5
Nicaragua	0.7	71.5	72.2	13.6	85.8
Uganda	0.4	68.9	69.2	16.2	85.5
Cambodia	0.1	64.6	64.7	9.0	73.7
Philippines	0.1	61.9	62.0	8.6	70.6
Iraq	0.2	60.9	61.2	8.0	69.1
Burundi	0.0	55.9	55.9	13.1	69.0
Benin	0.3	55.4	55.7	13.1	68.8
Cameroon	0.0	55.4	55.4	13.0	68.4
Madagascar	0.1	55.2	55.3	13.0	68.3

Country	Not research		Total country-specific	Regional allocation	Total including regional allocation
	Exclusively benefits PNH	Mentions PNH and others			
Eritrea	0.7	54.4	55.2	12.9	68.1
Bolivia	0.5	55.6	56.1	10.8	66.9
Liberia	0.0	53.4	53.4	12.5	66.0
Sudan	0.0	47.1	47.1	11.1	58.2
Ghana	2.0	44.2	46.3	10.9	57.1
Myanmar	0.7	49.3	50.0	6.5	56.5
West Bank and Gaza Strip	1.4	48.4	49.8	6.5	56.3
Senegal	0.8	43.9	44.7	10.5	55.2
Nepal	0.1	48.6	48.7	6.3	55.0
Somalia	0.5	43.0	43.5	10.2	53.7
China (People's Republic of)	0.1	45.5	45.6	6.4	52.0
Peru	0.0	39.6	39.6	7.6	47.3
Yemen	0.1	41.5	41.5	5.4	46.9
Sierra Leone	0.0	37.4	37.4	8.8	46.1
Guatemala	0.0	38.1	38.1	7.2	45.3
Rwanda	1.5	34.5	36.0	8.4	44.4
Chad	0.0	29.8	29.8	7.0	36.8
El Salvador	0.0	24.5	24.5	4.6	29.2
Niger	1.2	21.7	22.8	5.4	28.2
Georgia	0.1	21.7	21.8	3.9	25.7
Egypt	0.9	19.0	20.0	4.6	24.5
Central African Republic	0.0	17.5	17.6	4.1	21.7
Guinea	0.0	17.2	17.2	4.0	21.3
Congo	0.0	16.7	16.7	3.9	20.6
Lao People's Democratic Republic	1.2	16.6	17.8	2.5	20.3
Dominican Republic	0.2	16.1	16.3	3.7	20.0
Timor-Leste	0.1	16.8	16.8	2.3	19.2
Fiji	0.0	15.1	15.1	3.3	18.4
Tajikistan	0.3	14.5	14.9	2.7	17.5
Ukraine	2.9	12.3	15.3	2.2	17.4
Gabon	0.0	13.5	13.5	3.2	16.7
Côte d'Ivoire	0.0	13.0	13.0	3.1	16.1
Armenia	0.2	12.6	12.8	2.3	15.1
Sri Lanka	0.0	12.7	12.8	1.7	14.4
Moldova	0.2	12.3	12.5	1.8	14.3
Angola	0.3	11.3	11.6	2.7	14.3
Kyrgyzstan	0.1	11.0	11.1	2.0	13.1
Lesotho	0.0	10.6	10.6	2.5	13.1
Colombia	0.0	9.9	9.9	1.9	11.9

Country	Not research		Total country-specific	Regional allocation	Total including regional allocation
	Exclusively benefits PNH	Mentions PNH and others			
Paraguay	0.0	9.7	9.7	1.9	11.5
Uzbekistan	0.7	8.6	9.3	1.7	11.0
Belize	0.0	9.1	9.1	1.7	10.8
Guinea-Bissau	0.1	8.3	8.3	2.0	10.3
Panama	0.0	8.3	8.3	1.6	9.9
Democratic People's Republic of Korea	0.1	7.5	7.7	1.1	8.7
Costa Rica	0.0	7.3	7.3	1.4	8.7
South Africa	0.0	4.7	4.7	1.1	5.8
Albania	0.0	4.9	4.9	0.7	5.6
Syrian Arab Republic	0.1	4.6	4.8	0.6	5.4
Azerbaijan	0.1	4.3	4.4	0.8	5.1
Namibia	0.0	4.0	4.0	0.9	5.0
Mauritania	0.0	3.9	3.9	0.9	4.8
Nauru	0.0	3.8	3.8	0.8	4.6
Gambia	0.3	3.4	3.7	0.9	4.6
Ecuador	0.0	3.7	3.7	0.7	4.4
Kosovo	0.0	3.0	3.0	0.4	3.4
Argentina	0.2	2.5	2.7	0.5	3.2
Viet Nam	0.9	1.9	2.7	0.4	3.1
Serbia	0.2	2.5	2.7	0.4	3.1
Djibouti	0.0	2.2	2.2	0.5	2.7
Samoa	0.0	2.2	2.2	0.5	2.7
Togo	0.0	2.1	2.1	0.5	2.6
Brazil	0.2	1.9	2.1	0.4	2.5
Morocco	0.3	1.7	2.0	0.5	2.5
Mongolia	0.0	2.0	2.0	0.3	2.3
Papua New Guinea	0.0	1.8	1.8	0.4	2.2
Sao Tome and Principe	0.0	1.7	1.7	0.4	2.1
Turkmenistan	0.2	1.5	1.8	0.3	2.1
Bhutan	0.0	1.7	1.7	0.2	2.0
Kazakhstan	0.1	1.4	1.5	0.3	1.8
Tunisia	0.0	1.4	1.4	0.3	1.7
Iran	0.0	1.2	1.2	0.2	1.4
Comoros	0.0	0.9	0.9	0.2	1.1
Equatorial Guinea	0.0	0.9	0.9	0.2	1.1
Swaziland	0.0	0.9	0.9	0.2	1.1
Algeria	0.0	0.8	0.8	0.2	0.9
Venezuela	0.0	0.7	0.7	0.1	0.9
Lebanon	0.0	0.7	0.7	0.1	0.8
Maldives	0.0	0.6	0.6	0.1	0.7

Country	Not research		Total country-specific	Regional allocation	Total including regional allocation
	Exclusively benefits PNH	Mentions PNH and others			
Cuba	0.1	0.5	0.5	0.1	0.7
Turkey	0.0	0.5	0.6	0.1	0.6
Former Yugoslav Republic of Macedonia	0.0	0.4	0.5	0.1	0.5
Uruguay	0.3	0.0	0.3	0.1	0.4
Cabo Verde	0.0	0.3	0.3	0.1	0.4
Botswana	0.0	0.2	0.2	0.1	0.3
Mexico	0.0	0.2	0.2	0.0	0.3
Thailand	0.0	0.2	0.2	0.0	0.2
Mayotte	0.0	0.2	0.2	0.0	0.2
Palau	0.1	0.0	0.1	0.0	0.1
Jamaica	0.0	0.1	0.1	0.0	0.1
Marshall Islands	0.1	0.0	0.1	0.0	0.1
Libya	0.0	0.0	0.0	0.0	0.1
Mauritius	0.0	0.0	0.0	0.0	0.0
Belarus	0.0	0.0	0.0	0.0	0.0
Malaysia	0.0	0.0	0.0	0.0	0.0
Guyana	0.0	0.0	0.0	0.0	0.0
Bosnia and Herzegovina	0.0	0.0	0.0	0.0	0.0
Chile	0.0	0.0	0.0	0.0	0.0

Supplementary table 4: Regional funding mentioning PNH by recipient region

Total value of funding (constant 2013 USD, millions) disbursed to regional or unspecified recipients over the period 2003-13, by whether the funding exclusively benefitted PNH or also benefitted other population groups, and by whether the funding was for research or non-research activities. Regions are ranked from highest to lowest cumulative disbursements.

Region	Exclusively benefits PNH	Benefits PNH and others	Total region-specific
**TOTAL**	**8.2**	**1129.5**	**1137.7**
Bilateral, unspecified	4.7	717.3	722.0
South of Sahara, regional	2.8	186.1	188.9
Africa, regional	0.3	143.7	144.0
America, regional	0.1	38.4	38.4
Asia, regional	0.0	23.8	23.8
Central Asia, regional	0.0	3.9	3.9
Far East Asia, regional	0.0	3.4	3.4
Oceania, regional	0.0	2.2	2.3
North of Sahara, regional	0.0	1.4	1.5
Europe, regional	0.0	1.0	1.0
South & Central Asia, regional	0.0	0.7	0.7
South America, regional	0.0	0.4	0.4
Middle East, regional	0.2	0.1	0.3
South Asia, regional	0.0	0.1	0.1
North & Central America, regional	0.0	0.1	0.1
States Ex-Yugoslavia	0.0	0.0	0.0
West Indies, regional	0.0	7.1	7.1

Supplementary table 5: Results of regressions assessing the degree of targeting of PNH funding to health and economic need

The table presents output from our ordinary least squares regression analyses examining the degree to which official development assistance and private grants (ODA+) for prenatal and neonatal health (PNH) is targeted to health need (the neonatal mortality rate in 2008, nmr2008) and economic need (the natural logarithm of per capita gross domestic product in 2008, lnpcgdp2008). Both explanatory variables are centred by subtracting their mean values to facilitate interpretation. Analysis is conducted on those of the 156 countries in the Countdown ODA+ Database for which the necessary data is available. P-values that are statistically significant at the 5% level are highlighted in dark green and those that are statistically significant at the 10% level are highlighted in light green. We used Huber-White estimators to generate robust standard errors for all models.

Dependent variable	N obs	R-squared	Independent variables					
			lnpcgdp2008centred		nmr2008centred		interaction term	
			coefficient	p-value	coefficient	p-value	coefficient	p-value
Main analysis: y=Ln(funding)								
Total ODA+ mentioning PNH	118	0.4726	-1.4495	<0.001	0.0333	0.107	0.0436	0.007
ODA+ mentioning PNH per live birth	116	0.3463	-1.0301	<0.001	0.0327	0.058	0.0565	0.001
Total ODA+ exclusively benefitting PNH	95	0.0793	-0.7673	0.021	-0.0178	0.664	0.0181	0.474
ODA+ exclusively benefitting PNH per live birth	116	0.2943	-1.2609	<0.001	0.0491	0.054	0.0633	0.012
Alternative specification 1: y=Ln(funding), drop small countries from per birth								
ODA+ mentioning PNH per live birth	114	0.3573	-1.0138	<0.001	0.0359	0.037	0.0552	0.001
ODA+ exclusively benefitting PNH per live birth	114	0.2951	-1.2434	<0.001	0.0512	0.048	0.0618	0.014
Alternative specification 2: y=Ln(funding+1)								
Total ODA+ mentioning PNH	138	0.5244	-1.0213	<0.001	0.0199	0.180	0.0081	0.336
ODA+ mentioning PNH per live birth	136	0.3354	-0.5986	<0.001	0.0080	0.453	0.0156	0.02
Total ODA+ exclusively benefitting PNH	138	0.1493	-0.0734	0.032	0.0059	0.243	-0.0005	0.807
ODA+ exclusively benefitting PNH per live birth	136	0.1254	-0.3171	0.002	-0.0127	0.144	0.0051	0.322
Alternative specification 3: Y=Ln(funding+1), drop small countries from per birth								
ODA+ mentioning PNH per live birth	123	0.3183	-0.5576	<0.001	0.0097	0.347	0.0164	0.014
ODA+ exclusively benefitting PNH per live birth	123	0.1114	-0.3155	0.004	-0.0133	0.136	0.0060	0.292
Alternative specification 4: y=Ln(funding), excluding the extreme outlier specific to each model								
Total ODA+ mentioning PNH (EXCLUDE PAKISTAN, AFGHANISTAN)	116	0.4704	-1.5127	<0.001	0.0221	0.262	0.0505	0.001
ODA+ mentioning PNH per live birth (EXCLUDE INDIA)	114	0.404	-1.0723	<0.001	0.0377	0.027	0.0590	<0.001
Total ODA+ exclusively benefitting PNH (EXCLUDE BELIZE, FIJI)	94	0.084	-0.8166	0.015	-0.0279	0.494	0.0141	0.571
ODA+ exclusively benefitting PNH per live birth (NO OUTLIER)	NA							
